# Regeneration of multiple shoots from transgenic potato events facilitates the recovery of phenotypically normal lines: assessing a *cry*9Aa2 gene conferring insect resistance

**DOI:** 10.1186/1472-6750-11-93

**Published:** 2011-10-13

**Authors:** Sathiyamoorthy Meiyalaghan, Philippa J Barrell, Jeanne ME Jacobs, Anthony J Conner

**Affiliations:** 1 The New Zealand Institute for Plant & Food Research Ltd, Private Bag 4704, Christchurch 8140, New Zealand

## Abstract

**Background:**

The recovery of high performing transgenic lines in clonal crops is limited by the occurrence of somaclonal variation during the tissue culture phase of transformation. This is usually circumvented by developing large populations of transgenic lines, each derived from the first shoot to regenerate from each transformation event. This study investigates a new strategy of assessing multiple shoots independently regenerated from different transformed cell colonies of potato (*Solanum tuberosum *L.).

**Results:**

A modified *cry*9Aa2 gene, under the transcriptional control of the CaMV 35S promoter, was transformed into four potato cultivars using *Agrobacterium*-mediated gene transfer using a *npt*II gene conferring kanamycin resistance as a selectable marker gene. Following gene transfer, 291 transgenic lines were grown in greenhouse experiments to assess somaclonal variation and resistance to potato tuber moth (PTM), *Phthorimaea operculella *(Zeller). Independently regenerated lines were recovered from many transformed cell colonies and Southern analysis confirmed whether they were derived from the same transformed cell. Multiple lines regenerated from the same transformed cell exhibited a similar response to PTM, but frequently exhibited a markedly different spectrum of somaclonal variation.

**Conclusions:**

A new strategy for the genetic improvement of clonal crops involves the regeneration and evaluation of multiple shoots from each transformation event to facilitate the recovery of phenotypically normal transgenic lines. Most importantly, regenerated lines exhibiting the phenotypic appearance most similar to the parental cultivar are not necessarily derived from the first shoot regenerated from a transformed cell colony, but can frequently be a later regeneration event.

## Background

The development of transgenic plants to confer insect pest resistance is becoming a valuable component for integrated pest management (IPM) programmes [1]. Such genetic improvement of clonally propagated crops using a transgenic approach necessitates the recovery of the transgenic lines with the desired transgene expression coupled with retention of all the elite genetic attributes of the parental clone [2]. One of the major limitations to achieving this is the occurrence of 'off-types' resulting from somaclonal variation during the tissue culture phase of plant transformation [3,4]. Somaclonal variation is defined as genetic and phenotypic variation observed when plants are regenerated from cultured somatic cells [5-7]. Genotype, explant origin, cultivation period and the culture conditions are reported as four critical variables contributing to somaclonal variation [8]. The frequency of off-types attributed to somaclonal variation among populations of transgenic potatoes has been recorded as 15-80%, depending on the potato cultivar [9-15].

Reducing the frequency of these off-types during potato transformation is necessary to increase the likelihood of recovering transgenic lines equivalent to the parental clone with the beneficial effects from expression of the transgene [3]. This is important, since elimination of somaclonal variation via sexual hybridization cannot be achieved without simultaneously losing the genetic integrity of the potato clone. Asexual reproduction immediately fixes the initial hemizygous status of transgenes within the highly heterozygous genetic background of clonal cultivars. For this reason, transgenic potatoes are maintained as vegetative clones from the initial selection of the transformant in tissue culture through to commercial release [3]. We have recently described a new strategy to facilitate the recovery of phenotypically normal transgenic potato lines following transformation [4]. This involves the regeneration of multiple shoots from each transformation event. Marked differences in phenotypic variation were observed between these multiple regeneration events which must have originated after T-DNA insertion, and consequently during the tissue culture phase. This unequivocally demonstrated that somaclonal variation occurs during tissue culture and independent of transgene insertion. Furthermore, later regeneration events were more phenotypically normal than earlier shoots recovered from each transformation event, suggesting that reliance on only the first shoot regenerated may compromise the recovery of phenotypically normal transgenic lines [4].

The aim of the present work was to validate fully the strategy of regenerating multiple shoots from each transformation event to facilitate the recovery of phenotypically normal transgenic potato lines. Using a modified *cry*9Aa2 gene known to confer resistance to potato tuber moth (PTM), *Phthorimaea operculella *(Zeller) [16,17], we recovered multiple lines independently regenerated from numerous transformed cell colonies in four potato cultivars. All lines were assessed for the effectiveness of transgene performance and the appearance of somaclonal variation to test whether the first transgenic shoot regenerated from a transformation is the best performing transgenic clone.

## Methods

### Plant material

Virus-free plants of cultivars 'Iwa', 'Red Rascal', 'Karaka' and 'Pacific' were multiplied *in vitro *on a multiplication medium consisting of MS salts and vitamins [18], plus 30 g·l^-1 ^sucrose, 40 mg·l^-1 ^ascorbic acid, 500 mg·l^-1 ^casein hydrolysate, and 7 g·l^-1 ^agar [19]. The agar was added after pH was adjusted to 5.8 with 0.1 M KOH, then the medium was autoclaved at 121°C for 15 min. Aliquots of 50 ml were dispensed into (80 mm diameter × 50 mm high) pre-sterilized plastic containers (Vertex Plastics, Hamilton, New Zealand). Plants were routinely subcultured as two or three node segments every 3-4 weeks and incubated at 26°C under cool white fluorescent lamps (80-100 μmol·m^-2^·s^-1^; 16-h photoperiod).

### Transformation vector

**Figure 1:**
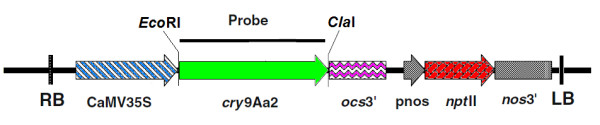
**The pART27G14 binary vector**. Schematic representation of the T-DNA region of the binary vector pART27G14 with the modified *cry*9Aa2 coding region under transcriptional control of the 35S promoter and the *ocs *3' region, plus a selectable marker gene conferring kanamycin resistance. The *Eco*RI and *Cla*I sites and the region used as a probe for Southern analysis are also illustrated. RB and LB represent the right and left T-DNA borders, respectively.

The modifications of the nucleotide sequence encoding the insecticidal moiety of the *cry*9Aa2 gene and the binary vector have been described and constructed previously [20]. The G14 version of this modified gene was the most effective against PTM in transgenic potato [17] and therefore used in this study. The pART27G14 binary vector (Figure 1) was transformed into *Agrobacterium tumefaciens *strain LBA4404 [21] using the freeze-thaw method [22].

### Development of transgenic plants

*A tumefaciens *culture harbouring the binary vector pART27G14 was incubated overnight in LB medium plus 300 mg·l^-1 ^spectinomycin at 28°C on a shaking incubator. Fully expanded leaves from *in vitro *potato plants were excised and cut in half across the midrib while submerged in the liquid *A. tumefaciens *culture. After about 30 s, these leaf segments were blotted dry on sterile filter paper (Whatman^® ^No. 1, 100 mm diameter). The leaf segments were then cultured, and transformed potato plants regenerated as previously described [23], except that transformed cell colonies were recovered on selection medium containing 100 mg·l^-1 ^kanamycin. When shoots regenerated from a cell colony, they were labelled as a series for each transformed cell colony, and transferred to multiplication medium containing 200 mg·l^-1 ^Timentin to suppress *A. tumefaciens *growth. To confirm kanamycin resistance in the resulting plants, individual shoots were subcultured onto medium containing 100 mg·l^-1 ^Timentin and 50 mg·l^-1 ^kanamycin, to eliminate any 'escapes' through the lack of root formation. Putative transgenic plants, from many transformed cell colonies, were transferred back to the multiplication medium containing only Timentin for clonal micropropagation.

### Screening of putative transgenic lines using PCR

Total genomic DNA from leaf tissue of *in vitro *plants was extracted based on a previously described method [24]. DNA was amplified in a multiplexed polymerase chain reaction (PCR) containing primers specific for the *cry*9Aa2 gene, the *npt*II gene, and the endogenous potato actin gene as an internal control (Table 1). PCR reactions were carried out in a Mastercycler^® ^(Eppendorf, Hamburg, Germany). Each 15 ul PCR mix contained 1x ThermoPol Reaction Buffer (20 mM Tris-HCl, 10 mM (NH_4_)_2_SO_4_, 10 mM KCl, 2 mM MgSO_4_, 0.1% Triton X-100, pH 8.8 at 25°C), 0.2 mM of each dNTP, 0.2 μM of each primer, 10-50 ng of template DNA and 0.5 U of Taq DNA Polymerase (New England BioLabs). The PCR profile was 2 min at 94°C, followed by 39 cycles of [30 s 93°C, 20 s 60°C, 70 s 72°C], finishing with 5 min at 72°C. Amplified products were separated by electrophoresis in a 2% agarose gel in 1x TAE buffer and visualized under UV light after staining with ethidium bromide.

**Table 1:** Primers for PCR of each gene and expected product size

Target gene	Forward primer (5' to 3')	Reverse primer (5' to 3')	Product size (bp)
*cry*9Aa2	GCACGGAATTATTGGCGCTTC	CACGATGTCCAACACCATCAA	424
*npt*II	ATTCGGCTATGACTGGGCACAACA	CCATGATATTCGGCAAGCAGGCAT	536
Actin	GATGGCAGAAGGCGAAGATA	GAGCTGGTCTTTGAAGTCTCG	1069

### Phenotypic evaluation of transgenic lines in a greenhouse

All PCR-confirmed transgenic lines were transferred to a containment greenhouse using the procedures and soil mix as previously described [12]. Two plants were established in each of three PB5 bags (15 cm × 15 cm × 15 cm black polythene bags) per line, with each PB5 bag treated as a replicate, and the bags placed in the greenhouse in a randomized block design. The greenhouse conditions provided heating below 15°C and ventilation above 22°C. Day length was supplemented to 16 h when needed with 500 W metal halide vapour bulbs, and relative humidity was maintained above 60%.

After 6-8 weeks in the greenhouse, the appearance of the foliage from each line was recorded using the following categories: phenotypically normal, marginal leaf curl, leaf wrinkling, reduced vigour, and/or stunted plants [12]. Tubers were also evaluated based on their size and appearance at the time of harvest, 14 weeks after planting in the greenhouse. Lines that produced only tubers < 10 mm in length (from apical to distal end) and/or were deformed in shape were considered abnormal.

### Insect bioassays using greenhouse-grown foliage

Leaf material from all transgenic lines with phenotypically normal shoots when grown in the greenhouse for at least 8 weeks was used for the PTM larvae bioassays. The insects used in the bioassays were obtained from a laboratory colony maintained as previously described [14]. The bottom of a 350 ml plastic container was lined with dry filter paper (Whatman^®^, No. 1, 50 mm diameter) into which were placed 3-5 terminal leaflets from the youngest, fully expanded leaves excised from plants of each replicate. Five neonate PTM larvae were weighed together, and then placed on the leaflets, after which the containers were sealed and kept in a controlled temperature room at 22 ± 3°C with a photoperiod of 16 h light: 8 h dark. Three replicates based on plants growing in separate PB5 bags were used for each line. The larvae were transferred to fresh leaflets 4 days later. The final weight for each surviving larva was recorded after 9 days. A growth index (GI) for each larva was calculated as GI = log_e _(final weight/mean initial weight). Transformed lines were screened for resistance to PTM over a period of 10 months in a series of 23 batches, all with the appropriate control (non-transgenic parental cultivar). The timing for testing a given line was dependent on when transformed lines were ready to be transferred to the greenhouse.

### Southern analysis

Genomic DNA was isolated from *in vitro *shoots of the kanamycin-resistant potato lines using Plant DNAzol (Invitrogen, Carlsbad, CA, USA) following the manufacturer's instructions. A total of ten micrograms of DNA per line was digested with *Eco*RI or *Cla*I, each enzyme restricting once within the T-DNA of the binary vector at either side of the *cry*9Aa2 coding region (Figure 1). The restricted DNA samples were used for Southern analysis as described previously [25] using Hybond N+ (Amersham, Uppsala, Sweden) membrane and probed according to the manufacturer's instructions. The probe used in the Southern analysis consisted of a fragment corresponding to the coding region of the *cry*9Aa2 gene labelled using a Megaprime DNA labelling system kit (Amersham Pharmacia Biotech, Piscataway, NJ USA) and α^32^P-dCTP.

### Statistical analysis

The data from batches of lines that had been screened for resistance to PTM at different times were analysed separately. Mean GI for each replicate for each line were analysed with analysis of variance. Comparisons with the control line were made as part of these analyses, and a probability level of 5% was used throughout to determine significance. Analyses were carried out using GenStat [26].

## Results

### Potato transformation

A total of 66, 64, 4 and 2 transformed cell colonies for Iwa, Red Rascal, Karaka and Pacific, respectively, were selected from leaf explants co-cultivated with *A. tumefaciens *harbouring a binary vector with a modified *cry*9Aa2 gene (pART27G14). Each putatively transformed cell colony was annotated with a number in one of five series (DG, SI, SK, SR, or SP) and a series of multiple shoots were independently regenerated from individual cell colonies. The first shoot regenerated from each cell colony was designated 'a', the second 'b', the third 'c', etc. In total 149, 110, 22 and 10 lines of Iwa, Red Rascal, Karaka and Pacific, respectively, were regenerated (Table 2). Only single shoots were regenerated from 30 cell colonies of Iwa and 39 cell colonies of Red Rascal. From the remaining cell colonies, the number of independently regenerated lines varied from two to eight (Table 3).

**Table 2:** Summary of transgenic potato lines

Cultivar	No. of lines PCR +ve for both *npt*II and *cry*9Aa2 genes	No. of lines with off-type foliage	No. of lines with abnormal tubers	No. of lines with phenotypically normal foliage used for PTM bioassay	No. of lines resistant to PTM larvae (*P *< 0.05)
'Iwa'	149	49	17	100	89
'Red Rascal'	110	28	42	82	76
'Karaka'	22	2	0	20	17
'Pacific'	10	0	0	10	9

Total	291	79	59	212	191

**Table 3:** Summary of the series of transgenic lines regenerated from individual cell colonies derived from cultivars 'Iwa' (DG and SI series), 'Red Rascal' (SR series), 'Karaka' (SK series) and 'Pacific' (SP series)

Transgenic series	Regenerated lines	Phenotypically normal lines	Lines with off-type foliage	Lines with abnormal tubers
'Iwa'				
DG1	a - h	a, h	-	b - g
DG2	a - d	d	-	a - c
DG3	a - f	a - f	-	-
DG4	a - f	a - f	-	-
DG5	a - b	b	-	a
SI1	a - b	b	a	-
SI2	a - b	-	b	a
SI3	a - b	a, b	-	-
SI4	a - c	c	a, b	-
SI5	a - b	a	b	-
SI6	a - b	a, b	-	-
SI9	a - g	a, c - g	b	-
SI10	a - b	a, b	-	-
SI12	a - b	b	a	-
SI15	a - e	a, c	b, d	e
SI16	a - h	a, d, f - h	b, c, e	-
SI17	a - d	d	a - c	-
SI18	a - d	d	a - c	-
SI20	a - d	d	a - c	-
SI21	a - b	-	a	b
SI22	a - c	-	a, c	b
SI23	a - b	-	b	a
SI24	a - b	b	a	-
SI25	a - c	c	a, b	-
SI27	a - g	a - g	-	-
SI28	a - b	a	b	-
SI34	a - b	b	a	-
SI35	a - c	c	a, b	-
SI40	a - b	b	a	-
SI43	a - b	b	a	-
SI48	a - b	b	a	-
SI49	a - b	-	a, b	-
SI50	a - c	c	a, b	-
SI51	a - b	b	a	-
SI52	a - b	b	a	-
SI56	a - c	c	a, b	-
'Red Rascal'				
SR1	a - b	a, b	-	-
SR4	a - b	a, b	-	-
SR5	a - h	h	d, f, g	a - c, e
SR7	a - b	b	-	a
SR8	a - c	c	-	a, b
SR10	a - d	d	-	a - c
SR12	a - d	d	c	a, b
SR13	a - c	c	b	a
SR17	a - c	c	-	a, b
SR18	a - b	b	a	-
SR19	a - f	f	c, e	a, b, d
SR20	a - b	-	a	b
SR21	a - b	a	b	-
SR22	a - b	-	-	a, b
SR23	a - c	c	a, b	-
SR28	a - b	b	a	-
SR29	a - b	b	a	-
SR34	a - b	b	a	-
SR35	a - d	d	a - c	-
SR36	a - b	b	a	-
SR37	a - c	c	a, b	-
SR40	a - b	b	-	a
SR44	a - b	-	b	a
SR45	a - b	b	a	-
SR52	a - b	-	b	a
'Karaka'				
SK1	a - f	a, c - f	b	-
SK2	a - g	a - g	-	-
SK3	a - f	b - f	a	-
SK4	a - c	a - c	-	-
'Pacific'				
SP1	a - d	a - d	-	-
SP2	a - f	a - f	-	-

### PCR analysis of regenerated lines

**Figure 2:**
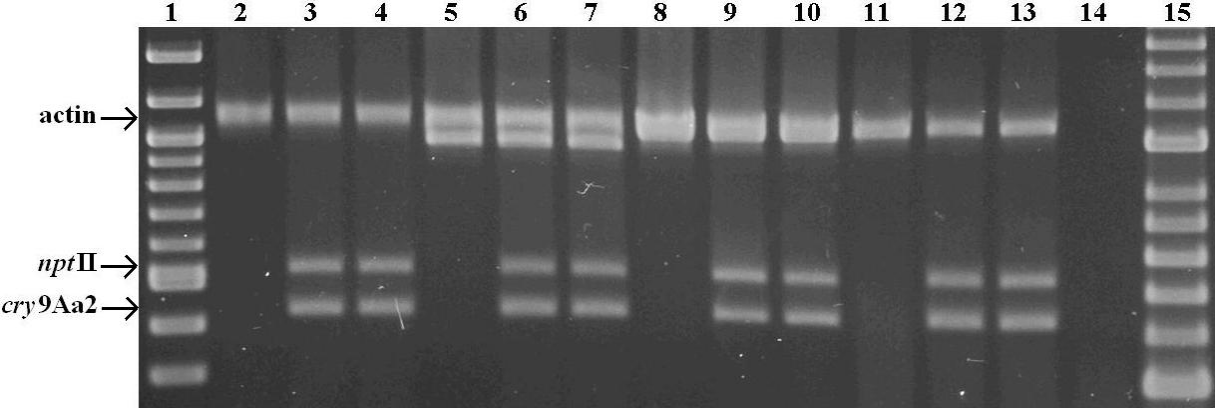
**PCR analysis of transgenic potato lines**. Lanes 1 and 15 are 100 bp molecular ruler N3231 (New England BioLabs) and HyperLadder™ II (Bioline), respectively; lanes 2-14 represent a multiplexed reaction with the *cry*9Aa2 primers producing an expected 424 bp product, the *npt*II primers producing an expected 536 bp product and the actin primers as an internal control producing one or two products depending on endogenous alleles (a 1069 bp product is expected in 'Iwa'); lanes 2, 5, 8 and 11, non-transgenic controls of cultivars 'Iwa', 'Red Rascal', 'Karaka' and ' Pacific', respectively; lanes 3-4 represent transgenic 'Iwa' lines; lanes 6-7 represent transgenic 'Red Rascal' lines; lanes 9-10 represent transgenic 'Karaka' lines; lanes 12-13 represent transgenic 'Pacific' lines; lane 14 is a no-DNA template control.

The presence of the *npt*II gene and the *cry*9Aa2gene in the regenerated lines was confirmed using multiplex PCR with an endogenous actin gene as an internal positive control. Since the actin product was expected in both transgenic and non-transgenic potato plants, this allowed a failed PCR reaction to be conveniently distinguished from a non-transgenic line. PCR products from representative lines are illustrated in Figure 2. All 291 putative transgenic lines were PCR positive for both the *npt*II and *cry*9Aa2 gene.

### Greenhouse evaluation and insect bioassays

From the 291 transgenic lines grown in the greenhouse experiments, 79 lines exhibited somaclonal variation with a range of off-type characteristics such as marginal leaf curl, leaf wrinkling, reduced vigour, abnormally small and/or deformed tubers, or a combination of these traits (Table 2). No PTM bioassays were conducted on these 79 lines since poor growth of PTM larvae may be a consequence of the abnormal foliage rather than the expression of the *cry*9Aa2 gene. Of the 212 lines used in the PTM bioassay, 59 lines yielded abnormally small and/or deformed tubers, despite having normal foliage appearance (Table 2).

**Figure 3:**
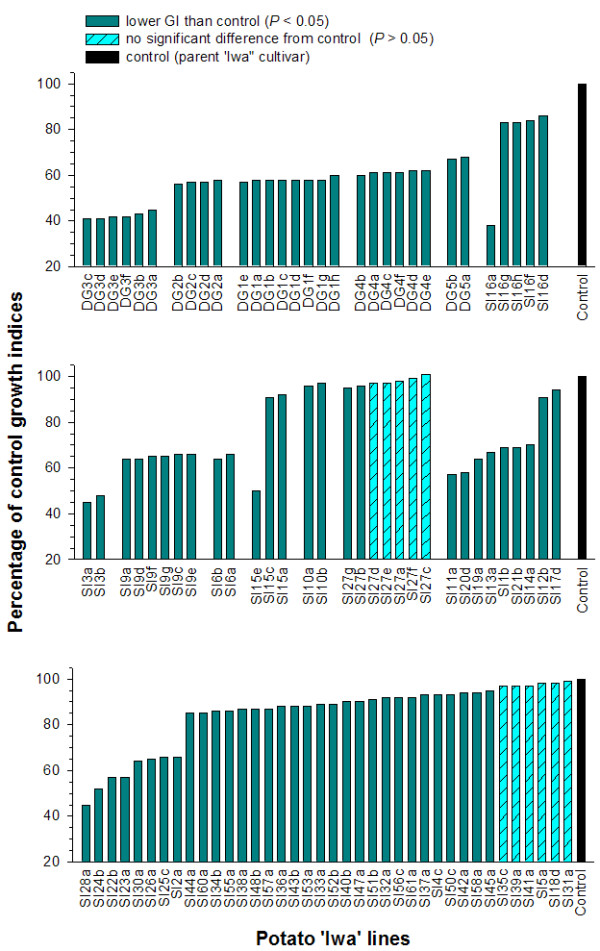
**Bioassays of insect resistance in potato cultivar 'Iwa'**. Growth indices (GI) of potato tuber moth (PTM) larvae fed foliage of 100 transgenic 'Iwa' potato lines expressed as a percentage of the larval GI on control foliage for the non-transgenic 'Iwa' potato. Where multiple regenerated lines from the same transformed cell colony were assessed, they are illustrated side-by-side.

**Figure 4:**
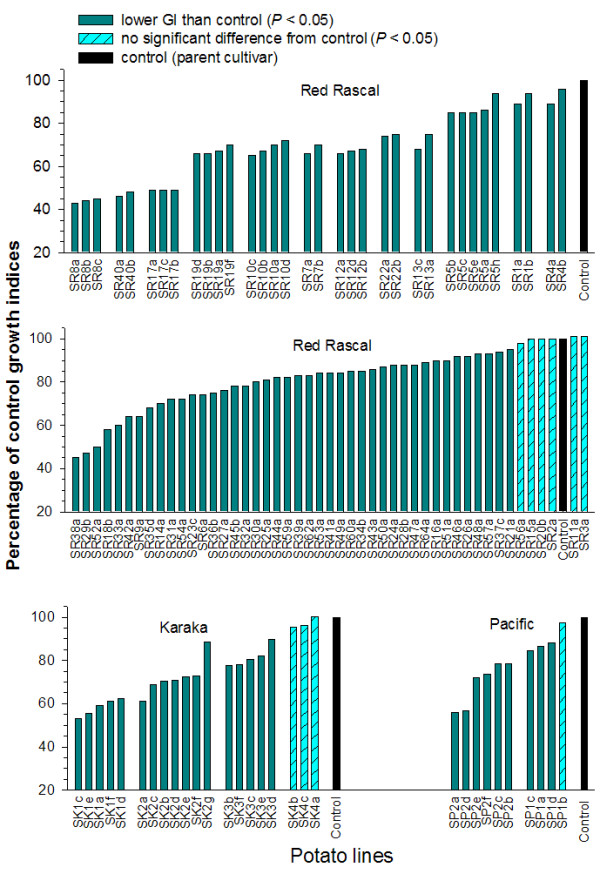
**Bioassays of insect resistance in potato cultivars 'Red Rascal', 'Karaka' and 'Pacific'**. Growth indices (GI) of potato tuber moth (PTM) larvae fed foliage of transgenic lines of potato cultivars 'Red Rascal', 'Karaka' and 'Pacific' expressed as a percentage of the larval GI on control foliage for the respective non-transgenic potato cultivars. Where multiple regenerated lines from the same transformed cell colony were assessed, they are illustrated side-by-side.

The GI of surviving PTM larvae relative to non-transgenic control plants is illustrated in Figures 3 and 4. The transgenic lines were tested as a series of batches of 8-10 lines, each with a non-transgenic control. The GI from each transgenic line is summarized as a percentage of the non-transgenic control plant in order to present the data in a simple and convenient manner and allow comparisons between the various batches. However, statistical analysis was performed on the original GI data. There was substantial variation in larval GI among the population of *cry*9Aa2-transgenic lines (Figures 3 and 4). The larval GI for 191 of the 212 lines was significantly lower than the non-transgenic controls, with the remaining 21 lines exhibiting no difference from the controls (Figures 3 and 4). In general, there was minimal variation among lines produced from the same transformation event. The only notable exceptions were the SI15 and SI16 series of transgenic lines. Lines SI15e and SI16a considerably inhibited larval growth compared with the remaining lines of SI16 and SI15 series transgenic plants (Figure 3). As expected, extensive leaf damage was observed in all the control potato lines where no resistance to PTM larvae was apparent. Substantially less leaf damage was observed on many of the lines transgenic for the *cry*9Aa2 gene.

### Southern analysis

**Figure 5:**
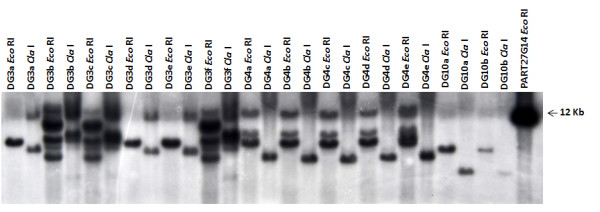
**Southern analysis of transgenic potato lines**. Southern analysis of selected potato lines transformed with the binary vector pART27G14 and probed with the coding region of the *cry*9Aa2 gene (Figure 1). Each plant DNA sample was digested separately with the enzymes indicated.

The transgenic status of *cry*9Aa2-transgenic lines was further confirmed by Southern analysis. Representative lines are illustrated in Figure 5. The majority of the regenerated lines from a single cell colony exhibited identical banding patterns and therefore confirmed as originating from the same transformed cell (e.g. DG4 a-e and DG10a-b). One exception was cell colony DG3 from which two groups of regenerated lines were recovered. Two distinct banding patterns were observed for lines DG3a, 3d, and 3e compared with plants DG3b, 3c, and 3f (Figure 5).

## Discussion

Analysis of all putative transgenic lines using PCR established the presence of the *npt*II and *cry*9Aa2 genes in all regenerated lines. This confirmed their transgenic status and a high rate of success for the *Agrobacterium*-mediated gene transfer system using kanamycin resistance as a selectable marker in potato [27]. Among the 212 transgenic lines tested for resistance to PTM larvae, 24 of them failed to exhibit improved resistance in the bioassay with excised leaves (Figures 3 and 4). This may be due to insufficient expression or accumulation of the Cry protein in the foliage of these transgenic lines to inhibit growth of PTM larvae. The remaining transgenic lines showed significantly lower GI than the non-transgenic control in PTM bioassays with excised leaves. Larvae recovered from these lines after the 9-day bioassay were small and of substantially lower weight. In our previous studies, PTM larvae with such poor growth in response to *cry *gene expression in transgenic potatoes have failed to reach pupation, resulting in complete disruption to the life cycle [14,16,28].

Considerable variation was observed in the level of PTM resistance among transgenic potato lines generated from independently derived cell colonies (Figures 3 and 4). However, PTM resistance observed within a series of transgenic plants derived from individual cell colonies (single transformation event) was minimal. Only two exceptions to this observation (in series SI15 and SI16) were evident from 30 transformation events for which multiple lines were assessed for insect resistance. Significant variation in transgene expression among insect-resistant transgenic plants from independent transgenic events has been commonly reported in other studies [29-31]. Such variation is usually attributed to unpredictable levels of transgene expression as a consequence of position effects resulting from differences in the integration site of the transgenes within plant genome and/or differences in T-DNA copy number [2]. The similar performance for insect resistance within a series of transgenic plants is expected since they are regenerated from the same cell colony, assumed to arise from a single transformation event.

Southern analysis of DNA fragments that encompass the integration site of transgenes in plant genomes provides a unique identifier for independent transformation events [4]. This approach was used to confirm whether multiple lines regenerated from the same transformed cell colony were derived from the same single cell or transformation event. This is illustrated in Figure 5, where the plant lines derived from the DG4 and DG10 series show identical hybridization patterns for both the left and right border fragments when probed with the inserted transgene. However, Southern analysis in the DG3 series confirmed that lines a, d and e were derived from a different transformation event from the lines b, c and f. This could happen when two cells adjacent to each other undergo random integration of T-DNA upon *Agrobacterium*-mediated transformation to form a chimeric cell colony from two independent transformation events. While the level of PTM resistance was consistent between these two sets of transgenic lines of the DG3 series, the differences observed within the SI15 and SI16 series are likely to result from more than one transformation event in the original selected cell colony.

The usual approach for producing transgenic cultivars in clonal crops involves the development of a large number of independently derived transgenic lines in order to recover several lines with the desired phenotype and transgene expression [2,3]. When developing large populations of independently derived transgenic lines, only the first shoot to regenerate from each transformation event is usually selected [14,17]. This is based on a widely held view that minimum time in tissue culture is favourable to avoid undesired somaclonal variation [32], and the assumption that a shoot taking a longer time to regenerate has a greater chance of producing off-types. In this study, a series of multiple lines were independently regenerated from 67 separate transformed cell colonies. In 37 of these series (55%), the first shoot recovered (suffix 'a' for each series) exhibited an off-type phenotype whereas a later shoot was phenotypically normal. Therefore, an important strategy for the genetic improvement of clonal crops involves the regeneration and evaluation of multiple shoots from each transformation event to facilitate the recovery of phenotypically normal transgenic lines.

## Conclusions

The regeneration of multiple shoots from the same transformed cell colony, coupled with Southern analysis, provided a means to generate and evaluate multiple transgenic potato lines from the same transformation event. The phenotypic performance of a *cry*9Aa2 transgene for insect resistance was similar for all lines independently regenerated from the same transformation event. However, these multiple lines frequently exhibited a markedly different spectrum of somaclonal variation, with the line exhibiting the phenotypic appearance most similar to the parental cultivar not being the first regenerated shoot for more than half of all transformation events. This study has confirmed the value of a new strategy to facilitate the recovery of phenotypically normal transgenic lines for the genetic improvement of clonal crops, which involves the regeneration and evaluation of multiple shoots from each transformation event.

## Authors' contributions

SM coordinated the data analysis and drafted the manuscript. PB coordinated the potato transformation, performed the Southern analysis, and helped to interpret the data and draft the manuscript. JJ participated in the design of the study, performed the PCR analysis, and helped to interpret the data and draft the manuscript. AC conceived the study, participated in its design, coordinated the insect bioassays, and helped draft the manuscript. All authors read and approved the final manuscript.
